# Surveillance of tuberculosis incidence and mortality through spatio-temporal analysis in Oyo State, Nigeria

**DOI:** 10.1371/journal.pone.0311739

**Published:** 2025-07-16

**Authors:** Titilade Kehinde Ayandeyi Teibo, Thais Zamboni Berra, Yan Mathias Alves, Reginaldo Bazon Vaz Tavares, Oluwaseyi Ademo Olayemi, Ricardo Alexandre Arcêncio

**Affiliations:** 1 Department of Maternal and Child Nursing and Public Health, University of São Paulo College of Nursing at Ribeirão Preto, Ribeirão Preto, São Paulo, Brazil; 2 Department of Physiology, Obafemi Awolowo University, Ile-Ife, Osun State, Nigeria; University of Uyo, NIGERIA

## Abstract

The study aimed to describe the epidemiological profile of Tuberculosis (TB) and analyze the spatial distribution and temporal trend of TB incidence and mortality in Oyo state Nigeria from 2015–2019. The study was composed of TB cases and deaths reported by the Oyo State Health Secretariat between 2015 and 2019. The purely spatial, space-time scanning and the Getis-Ord Gi* techniques were used to analyze the spatial characteristics of TB and to identify areas at risk for its occurrence and deaths based on rates in each unit of analysis - Local Government Area (LGA), while the time series analysis technique was used to assess the characteristics of TB in Oyo state over the period studied. The standardized rates of sex and age were considered for data analysis. We analyzed 28,670 new cases of pulmonary and extra pulmonary TB as well as 1142 deaths reported in all LGAs between 2015 and 2019. Ibadan South West local government had the highest rate and risk (334 cases per 100,000), solely constituting above 10% of all cases reported in all 33 LGAs and for deaths the rate was 19.01 cases/100,000 inhabitants. Ido and Oluyole LGAs were Hot Spot regions for TB with a 99% confidence interval (CI) for TB incidence; about TB mortality, five municipalities were identified with a spatial association at 95% CI. The historical series of TB incidence rate slightly increased between the years 2015–2017, with a sharp decline from then until 2019. The temporal trend for the incidence and mortality of TB in the period under study decreased. This study contributes to knowing the epidemiological profile, the spatial and temporal distribution, and areas with higher risk of TB transmission in Oyo state. This makes it possible for policy makers to target hotspot areas for intervention and disease prevention.

## Introduction

Tuberculosis (TB) spreads from person to person through the air. In their infectious state, when people with TB cough, sneeze, or spit, they expel the TB bacteria into the environment [1]. A person needs to inhale only a few of these germs to become infected. The disease is identified as the number one infectious killer disease in the world and among the top 10 causes of death worldwide. Approximately 5% of the world population is infected with TB without knowing because it is in its latent stage [2]. This is especially so because One-quarter of the World’s population, approximately 1.9 billion people, is infected with TB and symptoms appear early or late depending on individual immune competence [3].

Africa as a continent, is one that still has high TB burden despite several progress that has been made in the fight against TB over the years, this progress has suffered immense setbacks due to poor screening, and low accessibility of drugs and testing materials, socioeconomic determinants like undernutrition, diabetes and HIV co-infection also contribute to this setback [4].

Nigeria comes first in Africa and sixth globally among the high TB burden countries. having a TB burden of 219 cases per 100,000 population in 2020 [1]. Within the period of the COVID-19 pandemic alone, the country reported about 440,000 cases of TB [3] and the country still accounts for at least 300,000 missing cases every year. Nigeria is a high burden country for TB, multidrug resistant TB (MDR-TB), and TB-HIV [3]. The country has one of the lowest case detection rates among the high TB burden countries with only 117,320 (27%) of the incident cases being notified in 2019 [5]. This scenario inhibits infection control measures and leads to treatment initiation delay. In terms of epidemiology of TB, Nigeria ranks first in Africa.

Despite consistent interventions, the burden of TB in Nigeria is still high, as health facilities still lack consistent access to essential equipment and drug supplies. Also, diagnosis is not always easy [6]. Treatment takes several months and in a short while, loss of earnings for the sufferer may drive families into poverty, this multiplies the burden of the disease. In another vein, health workers do not receive fresh training and frequent supervision [7]. All these make up the big picture of the health problem in Nigeria.

Oyo state occupies the thirteenth (13th) position among states burdened with TB in Nigeria, the State TB control Program directs efforts towards creating awareness on the need to go for TB screening in government facilities. If there is a persistent cough for two weeks or more. Oyo state in the year 2021, recorded 11, 817 diagnosed and treated cases of TB. The state has five hundred and sixty-four (564) health facilities offering treatment for TB with ongoing services expansion to all parts of the State [6]. In the general context, Oyo State has 1,729 health facilities disaggregated into 712 Primary Health Centers (PHCs), 46 Secondary Health Facilities, 3 Tertiary Health Centers and 968 registered private health facilities [6].

A burden of 219 cases per 100,000 population is still far away from the 5 cases per 100,000 population targets envisioned by the WHO, The World Health Organization envisions a world free of TB, and its goals are to eradicate the disease by the year 2035 by reducing the number of deaths from TB by 95%, TB prevalence by 90% (i.e., fewer than 10 cases per 100,000 people), and to eliminate the disease’s catastrophic associated costs for households affected by the disease [7]. Hence a need to develop research to further investigate the situation of high TB burden in Oyo state and in Nigeria.

Therefore, the present study aims to describe the epidemiological profile of TB and analyze the spatial distribution and temporal trend of TB incidence and mortality in Oyo state Nigeria from 2015–2019.

## Materials and methods

### Study setting

**Fig 1:**
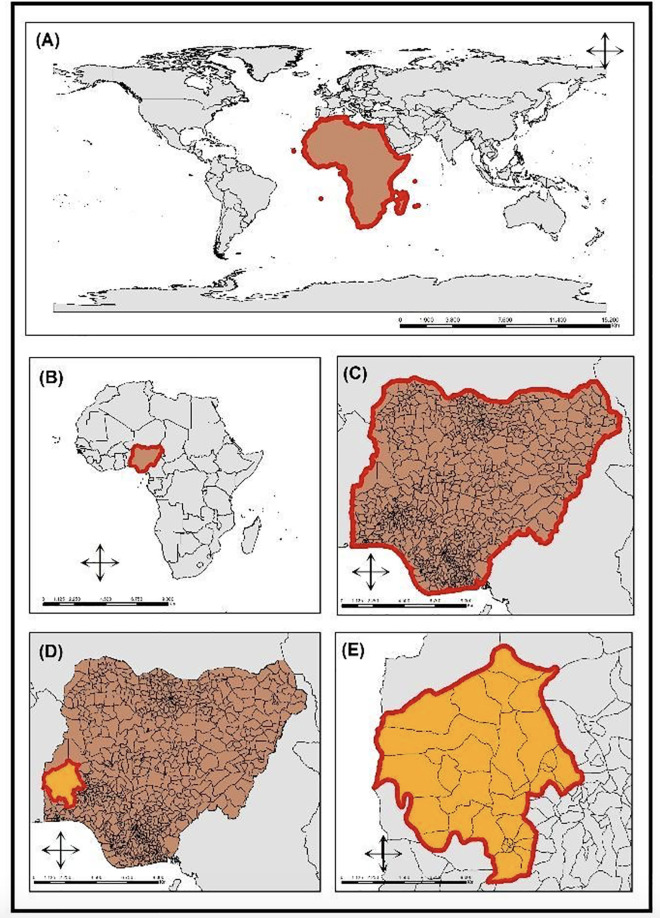
Geographic location of Oyo State, Nigeria. Legend: A) Geographical location of the African continent; B) Geographical location of Nigeria; C) Geographical division of Nigeria; D) Geographic location of Oyo state, Nigeria E) Geographic division of the LGAs of Oyo state. Source: Shapefile: Data Catalog – World Bank Group. Creation: Authors. Software: ArcGis version 10.5.

This is an ecological study that used the local government areas (LGAs) of Oyo State, southwestern Nigeria (Fig 1) as the unit of analysis, using a spatial and temporal approach.

**Fig 2:**
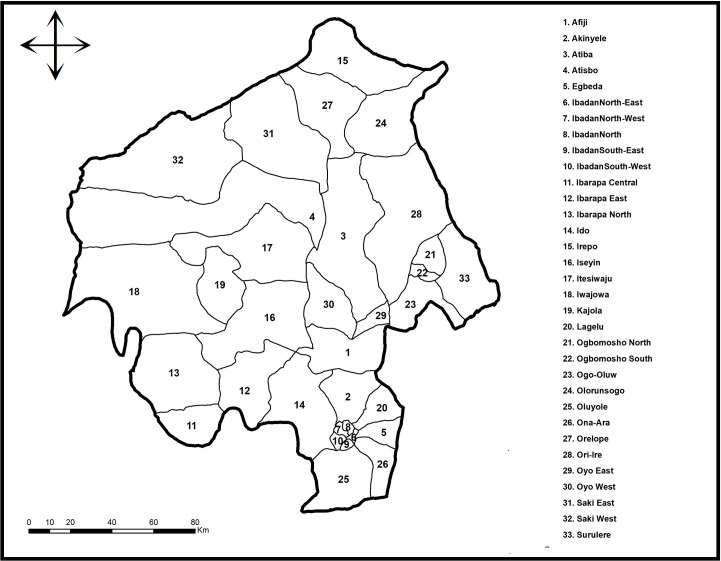
Geographic division of the LGAs of Oyo state, Nigeria. **Source**: Shapefile: Data Catalog – World Bank Group. Creation: Authors. Software: ArcGis version 10.5.

Oyo is made up of 33 LGAs, as can be seen in Fig 2 below.

Located in southwest Nigeria, Oyo State is an interior state. Ranking 13th position among the high TB states of Nigeria’s 36 states Ibadan, the country’s third-largest metropolis and formerly the continent’s second-largest city, serves as the state’s capital Kwara State, Osun State and Ogun State, all border Oyo State on its northern, eastern, and southwestern borders, respectively [8]. The state has an estimated population of 7,840,864; it is rated 6^th^ in terms of population and 14th in terms of size in Nigeria and has an area of about 28,454 squares. The area is located at 3.9470°E longitude and 73775°N latitude. For our study, individual Local government areas in Oyo state were the units of measurement for spatio-temporal analysis.

TB diagnosis is coordinated by the NTBLCP (National Tuberculosis and Leprosy Control Program) which is structured along the three tiers of government, i.e., Federal, State and Local Government Area [5]. The majority of TB services are offered as a part of primary healthcare, which is then followed by secondary and tertiary care offered by governmental and commercial institutions. Consultations, tests, medications, and hospitalization care for TB are all free in the public sector. Supervised TB treatment, health education and adherence, counseling, as well as HIV counseling and testing, remain the main objectives of the NTBLCP Offering free Directly Observed Treatment Short Course (DOTS) to all people with active TB [8]. The Yoruba ethnic group mainly inhabits the state. The climate is equatorial with dry and wet seasons and relatively high humidity.

### Population, information sources and selection criteria

The study population consisted of retrospective data of TB cases and deaths notified from January 1, 2015, to December 31, 2019, through the Oyo state health secretariat – yearly report obtained and collated manually by the state- level health service officers. TB data is usually collected from the Local government area health service, transferred to the state health secretariat, and collated in the quarterly health report in Nigeria. Data was accessed in 01/02/2021 by the authors and was available as Supplementary Material (S1 Table). Written informed consent of ethical approval was granted for this work approved by the Oyo state research and ethics committee board, available as Supplementary Material (S1 File).

### Data extraction, inclusion and exclusion criteria

The data extracted were the number of cases and deaths from TB reported in Oyo, according to LGA. As inclusion criteria, all cases of TB with a confirmed bacteriological diagnosis were considered, considering all age groups. Only deaths that had TB as their underlying cause were included. Presumptive cases of TB or cases without diagnostic confirmation were not included in the study, nor were deaths that had other underlying causes or TB as a secondary cause.

### Data analysis

First, the incidence and mortality standardized rates by sex and age were calculated. This procedure was performed using RStudio version 3.3.0 software (RStudio Inc., Boston, MA, USA).

The shapefile of the Oyo LGAs was obtained free of charge through the website of the Nigerian ministry [9] and, with the previously calculated incidence and mortality rates, this tabulated data was joined to the spatial data shapefile using the ArcGis software version 10.5. This process of transforming tabulated data into a specific reference system used in the shapefile is called georeferencing, which is the step that precedes data analysis, which is called geoprocessing [10]. The final file followed the UTM projection and Geocentric Reference System for the Americas (SIRGAS) 2000 datum.

Using natural break classification (Jenks), rates were grouped based on natural patterns inherent in the data. Class breaks are created to group similar values together and maximize differences between classes. Features are divided into classes whose boundaries are defined where there are relatively large differences in data values, providing better visualization and allowing areas with different levels of disease incidence and mortality to be clearly identified. By maximizing differences between classes, the Jenks method facilitates the detection of geographic patterns and trends, aiding in informed decision-making and the implementation of more effective public health policies.

To determine the existence of global and local spatial association between TB incidence and mortality rates, the Getis-Ord General G and Getis-Ord Gi* technique was used, respectively, using the rates calculated previously (incidence and mortality)

The Getis-Ord General G technique is based on the Moran Global Index and, as in inferential statistics, the results are based on the null hypothesis that there is no spatial clustering. If the p-value is significant, the null hypothesis can be rejected and the z-score value becomes important, in which its values of +/-3 represent a confidence level of 99% [11] If the z-score value is positive, the observed G Index is higher than expected, indicating high rates of the event clustered in the study area. If the z-score value is negative, the observed G Index is lower than the expected index, indicating that the low values are clustered in the study area [11].

To determine the existence of local spatial association between TB incidence and mortality rates, the Getis-Ord Gi* technique was used. In the Getis-Ord Gi* analysis, there is also a *z score* for each unit of analysis. The higher the *z-score*, the more intense the clustering of high values (Hotspot) (hotspots are areas of high incidence/prevalence, having high degree of disease transmissibility, with greater possibility for the emergence of a disease) [12], while the lower the *z-score* value, the more intense the clustering of low values or the lower occurrence of the event (Cold spot) [13].

In addition to the z-score, the analysis provides the p-value and the bin confidence level (Gi-Bin). Gi-Bin values identify statistically significant hot and cold spots, so results can be found as +3 (hotspots at 99% confidence level), + 2 (hotspots at 95% confidence level), + 1 (hotspots with a 90% confidence level), zero (when the unit of analysis does not show statistical significance, i.e., p-value > 0.05), −1 (coldspots with a 90% confidence level), – 2 (coldspots with a 95% confidence level) or −3 (coldspots with a 99% confidence level) [11].

To perform the Getis-Ord General G and G* technique, we used the LGAs of Oyo as the unit of analysis and the incidence and mortality rates for TB were entered as input data, separately, with a bandwidth stipulated by the tool called Incremental Spatial Autocorrelation (ISA), present in the Arcgis software version 10.5 [11]. This tool is also based on the Moran Index, where a series of increasing distances are tested, measuring the intensity of spatial clustering for each distance, and providing its Z score, which reflects the intensity of spatial clustering. The radius (or bandwidth) was considered the first statistically significant distance peak, indicating the distance where the spatial processes that promote clustering are most pronounced [11].

The Getis-Ord Gi* analysis and the choropleth map with the representation of areas identified with the presence of spatial association for TB incidence and mortality rates were also performed using ArcGis software version 10.5.

For risk cluster detection, the purely spatial scanning statistical technique developed by Kulldorff and Nagarwalla (1995) [14] also known as Scan statistics, was performed using SatScan software, version 9.6. Risk clusters were identified through circular windows, with a variable radius around the centroid of each census sector in the LGA under analysis. For this study, each window tested our formal hypothesis, i.e., H0: there are no spatial clusters in LGAs of Oyo State, and H1: in a certain area, the probability of occurrence of TB cases or deaths is lower or higher than in other LGAs of Oyo State.

For the identification of spatial clusters, the following criteria were used in the analysis software: Poisson’s discrete model, the clusters cannot be geographically overlapped, maximum size of the clusters equal to 6% of the exposed population (defined by the Gini index indicated by the analysis software itself), clusters with circular shape and 999 replications following the Monte Carlo criteria [15].

The Relative Risk (RR) of each identified cluster was calculated which is the probability of the event (TB cases or deaths) occurring in the exposed group (cluster) compared to the unexposed group (outside the cluster). For interpretation purposes, RR = 1 means that there is no statistically significant difference between the exposed and unexposed groups; if RR < 1, this tends towards zero, i.e., low risk (or protection), whereas RR > 1 denotes a risk area. Clusters with a *p-value* < 0.05 were considered statistically significant, and the 95% confidence interval (CI) was estimated.

Scanning statistics also made it possible to incorporate temporal factors, in which the interest is in the identification of event clusters, in the case of this research, cases of TB, which have occurred in space and time, simultaneously. Thus, the same criteria mentioned above were used in the analysis software adding precision of time in year and time period between 2015 and 2019. RR was also calculated for the clusters identified in the space-time analysis.

Finally, to verify the trend of incidence rates and mortality over the period under study, first, the rates (incidence and mortality due to TB) were calculated per year (2015–2019). We used the decomposition method called Seasonal and Trend Decomposition with Loess (STL), which is based on a locally based regression [16] to estimate the temporal tendency in the period under study.

STL is a robust time series decomposition method that uses locally adjusted regression models to decompose a time series into its components, namely, seasonality and noise (remaining data not explained by trend or seasonality, usually random) [17].

The STL method is robust, as it is a time series decomposition method that utilizes locally fitted regression models to separate the original series into three main components: trend, seasonality, and residuals (remaining data unexplained by trend or seasonality, typically random) [16].

The STL method performs smoothing on the time series using LOESS in two loops; the inner loop iterates between seasonal and trend smoothing, and the outer loop minimizes the effect of outliers. During the inner loop, the seasonal component is calculated first and removed to compute the trend component. The residuals are then calculated by subtracting the seasonal and trend components from the time series [16,17].

The three components of STL analysis relate to the raw time series as follows:


$${\text{y}}_{\text{i}}{\text{\textbackslash{} = \textbackslash{} s}}_{\text{i}}{\text{\textbackslash{} + \textbackslash{} t}}_{\text{i}}{\text{\textbackslash{} + \textbackslash{} r}}_{\text{i}}$$

where:

y_i_ = The value of the time series at point i.

s_i_ = The value of the seasonal component at point i.

t_i_ = The value of the trend component at point i.

r_i_ = The value of the remaining component at point i.

It is valid to mention that the seasonal component of an STL output reveals the recurrent temporal pattern present in the data based on the chosen seasonality, where if there’s a seasonal pattern, it typically takes the form of an oscillating or wave pattern. The trend component is the second component calculated during the inner loop, where the values of the seasonal component are subtracted from the raw data, eliminating the seasonal variation from the time series, and a smoothed trend line is then created by applying LOESS to the remaining values. Finally, the component pertaining to the residuals is calculated by subtracting the values of the seasonal and trend components from the time series, where the remaining values indicate the amount of noise present in the data, such that values close to zero indicate that the seasonal and trend components accurately describe the time series, while larger remaining values indicate the presence of noise [16,17].

There are several methods for time series decomposition, such as simple linear models, joint point models, and Generalized Additive Models (GAM). However, the STL method offers several advantages compared to other techniques, highlighting flexibility in detecting nonlinear patterns, robustness in the presence of outliers, suitability for time series with irregular seasonality, ability to handle changes in trend and seasonality over time, and interpretability of decomposed components. In summary, STL is a versatile and robust technique for time series decomposition, especially in scenarios where data exhibit nonlinear patterns, irregular seasonality, or changes in trend over time. It offers a combination of flexibility, robustness, and interpretability that makes it an attractive choice in many time series analysis contexts [16,17].

For the analysis execution, the forecast package [18] was used, where it is necessary to define some parameters: Period (seasonality period in the time series, for example, if the data is monthly and exhibits annual seasonality, the period would be 12); Window (size of the LOESS smoothing window used to estimate the trend component – a larger window will result in a smoother trend, while a smaller window will capture short-term variations); Inner Window and Outer Window (seasonality windows control the smoothing applied to the seasonal component, where the inner window is used to smooth seasonality, while the outer window is used to smooth trend); Robustness Iterations (determines the number of iterations in the robustness algorithm, so that the STL method can be adjusted to be robust against outliers); and finally Spline Degree (defines the degree of the polynomial used in the LOESS fit, where a higher degree allows for more flexible fits, while a lower degree results in smoother fits) [18].

Another important aspect to mention is that in the STL method, there is no fixed value for “p” or “regression coefficient,” as in traditional models since these terms are not specifically used in this context. However, each of these mentioned parameters can be adjusted to balance the smoothing of trend and seasonality components, as well as the method’s ability to adapt to the specific characteristics of the time series in question [16–18].

The graphics with the historical series and estimated time trend were performed using RStudio version 3.3.0 software (RStudio Inc., Boston, MA, USA).

Ethical approval for the present research was obtained from the Oyo State health secretariat.

## Results and discussion

**Fig 3:**
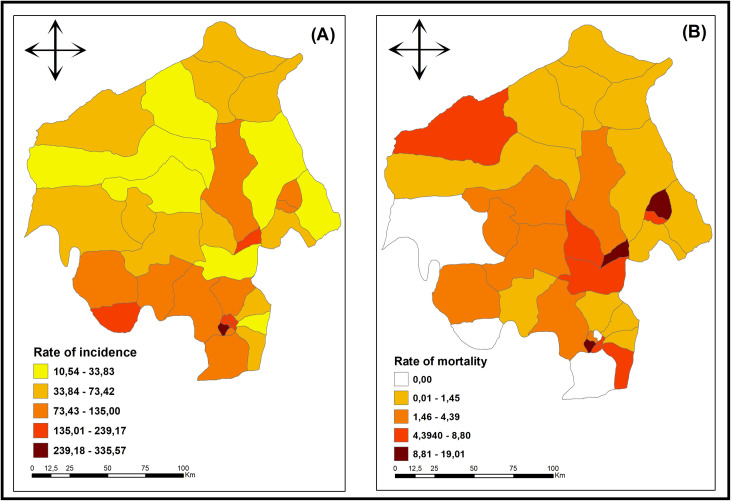
Distribution of tuberculosis incidence and mortality rates (per 100 thousand inhabitants), Oyo – Nigeria (2015 - 2019). **Legend**: A) Distribution of tuberculosis average incidence rates according to LGAs in Oyo state, Nigeria (2015 - 2019); B). Distribution of tuberculosis average mortality rates according to LGAs in Oyo state Nigeria (2015 - 2019). **Source**: Data: Ministry of Health of Nigeria; Shapefile: Data Catalog – World Bank Group; Creation and analysis: Authors; Software: ArcGis version 10.5.

During the study period (2015–2019), 28,680 TB cases and 1,131 TB deaths were reported. The highest incidence rate was observed in Ibadan South West LGA (334 cases/100,000 inhabitants) and the lowest rate was observed in Surulere LGA (9 cases/100,000 inhabitants) with high and low relative risk respectively (Fig 3A). As for the mortality rate, the highest rate was observed in the Oyo East Local government Area (19.01 cases/100,000 inhabitants) with a high RR and the lowest rates were observed in Ibadan North, Ibarapa Central, Iwajowa and Oluyole LGAs with low RR, with no deaths from TB in the period under study, according to (Fig 3).

Using the Getis-Ord General G technique, the existence of a spatial association was demonstrated for both the incidence of TB (Observed General G: 0.000024; z-score: 4.708469 and p-value: < 0.001) and mortality (Observed General G: 0.000012; z-score: 3.425193 and p-value: < 0.001), indicating the existence of a hotspot for the aforementioned rates in Oyo state.

**Fig 4:**
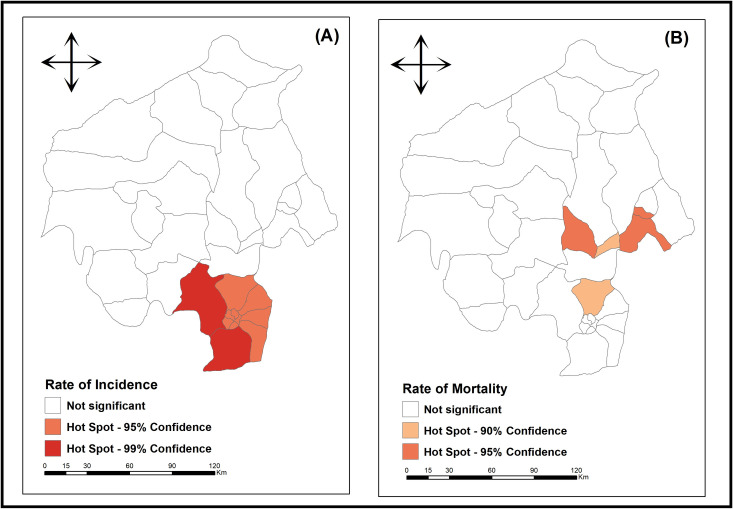
Areas with spatial association for tuberculosis incidence and mortality, Oyo – Nigeria (2015 - 2019). **Legend:** A) LGAs with spatial association for tuberculosis average incidence rate in Oyo state, Nigeria (2015 - 2019); B) LGAs with spatial association for tuberculosis average mortality rate in Oyo state, Nigeria (2015 - 2019). **Source**: Data: Ministry of Health of Nigeria; Shapefile: Data Catalog – World Bank Group; Creation and analysis: Authors; Software: ArcGis version 10.5.

Using the Getis-Ord Gi* technique for TB incidence rates, 11 municipalities with spatial association were identified, with Ido and Oluyole classified as HotSpot (areas of high incidence/prevalence, having high degree of disease transmissibility, with greater possibility for the emergence of a disease) [13] and presenting 99% confidence the municipalities Akinyele, Lagelu, Egbeda, Ona -Ara, Ibadan North-West, Ibadan South-West, Ibadan South-East, Ibadan North-East and Ibadan North are also classified as HotSpot, but with 95% confidence (Fig 4A).

As for TB mortality rates, using the Getis-Ord Gi* technique, five municipalities were identified with a spatial association for the event, with Oyo West, Ogo-Oluwa and Ogbomoso South classified as Hot Spot and presenting 95% confidence and the Oyo East and Akinyele municipalities also classified as HotSpot, but with 90% confidence (Fig 4B).

It is important to mention that for incidence rates, no clusters classified as + 1 (hotspots with a 90% confidence level) were found, and for mortality rates, no clusters classified as +3 (hotspots at 99% confidence level) were found. For both indices, no coldspots were found.

**Fig 5:**
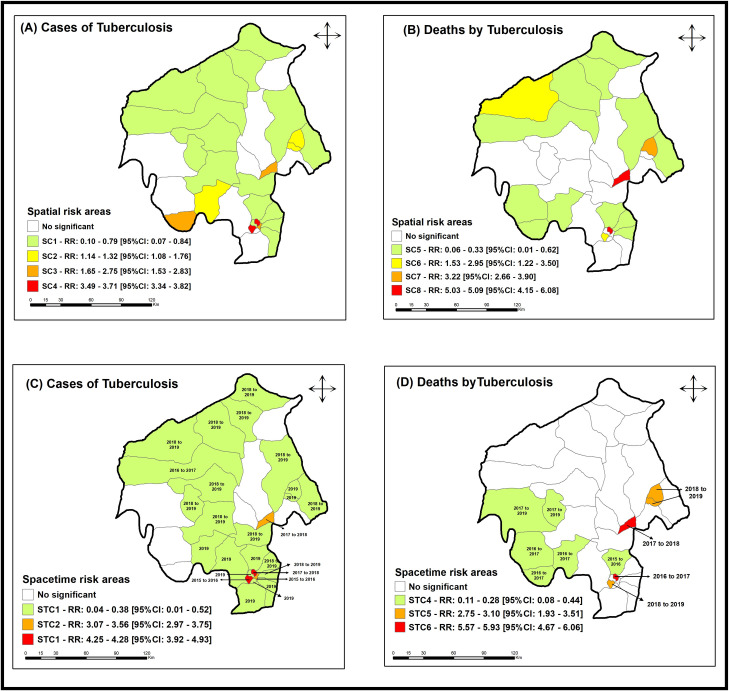
Areas with spatial risk for tuberculosis cases and deaths, Oyo – Nigeria (2015 - 2019). **Legend:** A) LGAs with spatial risk for tuberculosis cases in Oyo state, Nigeria (2015–2019); B) LGAs with spatial risk for tuberculosis deaths in Oyo state, Nigeria (2015–2019). **Source:** Data: Ministry of Health of Nigeria; Shapefile: Data Catalog – World Bank Group; Creation and analysis: Authors; Software: ArcGis version 10.5.

With the application of the purely spatial scanning technique, it was possible to identify a statistically significant cluster (p < 0.01) in the period corresponding to the years 2015–2019 for TB cases and deaths (Fig 5A and Fig 5B), corroborating the alternative hypothesis that there are areas in the municipality with a higher risk for the occurrence of TB in LGAs of Oyo State.

The spatial cluster 1 (SC1), considered as a protection for the event, presented an RR: 0.10 to 0.79 (95%CI: 0.07–0.84), formed by 16 LGAs (Egbeda, Orelope, Irepo, Saki East, Surulere, Ori-Ire, Itesiwaju, Kajola, Saki West, Atisbo, Afijio, Iseyin, Lagelu, Ibadan North-West, Ona-Ara and Akinyele), with a population of 2,870,865 people, 14,748 expected cases, and 6,864 observed cases.

The spatial cluster 2 (SC2) with RR: 1.14 to 1.32 (95%CI: 1.08–1.76), was composed by three LGAs (Ibarapa East, Ogbomosho North and Ogbomosho South), with a population of 416,420 people, 2,139 expected cases, and 2,542 observed cases.

The spatial cluster 3 (SC3), presented RR: 1.65 to 2.75 (95%CI: 1.53–2.83), was composed by three LGAs (Ibadan North-East, Oyo East and Ibarapa Central), with a population of 519,663 people, 2,669 expected cases, and 3,409 observed cases.

Finally, spatial cluster 4 (SC4), with an RR: 3.49 to 3.71 (95%CI: 3.34–3.82), was composed by two LGAs (Ibadan South-West and Ibadan North), with a population of 437,127 people, 2,245 expected cases, and 7,338 observed cases.

Applying purely spatial scanning statistics to TB deaths in Oyo State, it was also possible to find risk and protection clusters for the analyzed event. The spatial cluster 5 (SC5) (Fig 5B), considered as a protection for the event, presented an RR: 0.06 to 0.33 (95%CI: 0.01–0.62), formed by 15 LGAs (Ibadan North-West, Oluyole, Iwajowa, Egbeda, Orelope, Irepo, Saki East, Surulere, Ibarapa Central, Ibarapa North, Ibarapa East, Akinyele, Ori-Ire, Atisbo and Lagelu), with a population of 2,670,171 people, 530 expected cases, and 70 observed cases.

The spatial cluster 6 (SC6) with RR: 1.53 to 2.95 (95%CI: 1.22–3.50), was composed by two LGAs (Saki West and Ibadan South-West), with a population of 556,366 people, 110 expected cases, and 232 observed cases.

The spatial cluster 7 (SC7), presented RR: 3.22 (95%CI: 2.66–3.90), was composed of one LGA (Ogbomosho North), with a population of 198,859 people, 39 expected cases, and 118 observed cases.

Finally, spatial cluster 8 (SC8), with an RR: 5.03 to 5.09 (95%CI: 4.15–6.08), was composed by two LGAs (Oyo East and Ibadan North), with a population of 407,193 people, 80 expected cases, and 265 observed cases.

With the application of the space-time scanning technique, it was possible to identify a statistically significant cluster (p < 0.01) in the period corresponding to the years 2015–2019 for TB cases and deaths (Fig 5C e Fig 5D). The space-time cluster 1 (STC1), considered as a protection for the event, presented an RR: 0.06 to 0.38 (95%CI: 0.01–0.52), formed by 22 LGAs (Surulere, Ibadan North-West, Ori-Ire, Iseyin, Afijio, Egbeda, Atisbo, Ibarapa East, Ido, Itesiwaju, Kajola, Ona-Ara, Akinyele, Saki West, Orelope, Irepo, Saki East, Ibadan South-East, Oluyole, Ogbomosho North, Ogbomosho South and Lagelu), with a population of 4,014,095 people, 5793 expected cases, and 1291 observed cases.

The space-time cluster 2 (STC2) with RR: 3.07 to 3.56 (95%CI: 2.97–3.75), was composed by Oyo East and Ibadan North-East, with a population of 278,124 people, 571 expected cases, and 2070 observed cases.

The space-time cluster 3 (STC3), presented RR: 4.25 to 4.28 (95%CI: 3.92–4.93), was composed of Ibadan North and Ibadan South-West, with a population of 437,127 people, 898 expected cases, and 3637 observed cases.

Applying space-time scanning statistics to TB deaths in Oyo State, it was also possible to find risk and protection clusters for the analyzed event (Fig 5D). The space-time cluster 4 (STC4), considered as a protection for the event, presented an RR: 0.11 to 0.28 (95%CI: 0.08–0.44), formed by 15 LGAs (Ibadan North-West, Oluyole, Iwajowa, Egbeda, Orelope, Irepo, Saki East, Surulere, Ibarapa Central, Ibarapa North, Ibarapa East, Akinyele, Ori-Ire, Atisbo and Lagelu), with a population of 2,447,980 people, 183 expected deaths, and 171 observed deaths.

The space-time cluster 5 (STC5) with RR: 2.75 to 3.10 (95%CI: 1.93–3.51), was composed by Saki West, Ibadan South-West and Ogbomosho North, with a population of 932,102 people, 63 expected deaths, and 111 observed deaths.

Finally, space-time cluster 6 (STC6), with an RR: 5.57 to 5.93 (95%CI: 4.67–6.06), was composed by Oyo East and Ibadan North, with a population of 278124 people, 22 expected deaths, and 121 observed cases.

**Fig 6:**
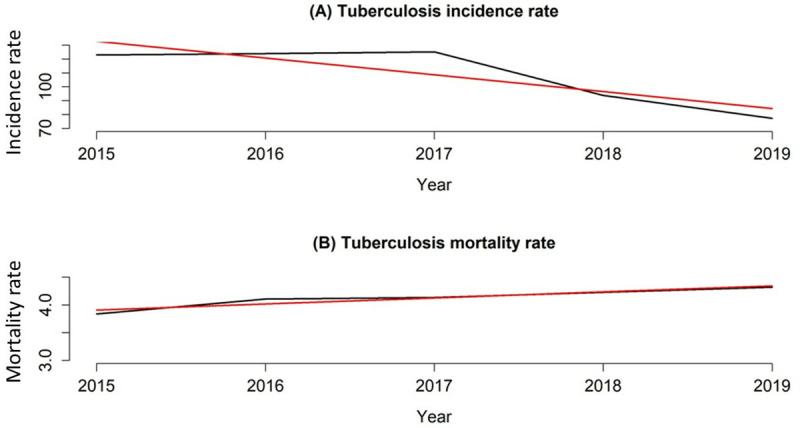
Series and time trend of tuberculosis incidence and mortality rates, Oyo – Nigeria (2015 - 2019). **Legend:** A) Series (black line) and time trend (red line) of tuberculosis incidence rate in Oyo state, Nigeria (2015–2019); B) Series (black line) and time trend (red line) of tuberculosis mortality rate in Oyo state, Nigeria (2015–2019). **Source**: Data: Ministry of Health of Nigeria; Creation and analysis: Authors; Software: RStudio version 3.3.0.

Finally, using the time series and the STL technique to estimate the temporal trend of this study, it is possible to observe in (Fig 6A) that the historical series of the TB incidence rate showed a slight increase between the years 2015–2017, while it showed a sharp decline until 2019. It is also possible to notice that the temporal trend in the incidence of TB in the period under study was decreasing, with average annual percentage change (APC) was −10.45%.

While in (Fig 6B) we see a slight increase in the TB mortality rate throughout the analyzed period, we can also see that the temporal trend of the event between 2015 and 2019 is increasing. During the period analyzed, the temporal trend in TB deaths in Oyo was classified as increasing with APC: + 2.04%/year. The components of the decomposed time series can be seen as Supplementary Material (S1 Fig).

Regarding gender and TB, throughout the study period, there was a higher number of TB cases among Males compared to females and the lowest TB incidence rate was seen in the 0–4 age group while the highest incidence rate was seen in the age group between 35–44. The profile of the age group and gender can be found in the Supplementary Material (S2 Table).

The spatial analysis and time series of TB is a technique that is still less commonly used in Nigeria and in Oyo state specifically in recent years. Nigeria had an incidence rate of 219/100,000 population in 2016 [19], the LGA with the highest incidence rate from our study- Ibadan southwest has a rate of (334 cases/100,000 population) which is higher than that of the entire country earlier cited. Being very variable and heterogeneous, the LGA with the lowest rate- Surulere has 9 cases/100,000 inhabitants.

This evidence the existence of risk variability within the same state. For certainty, this local government area with the highest incidence rate is reputed to be the most potentially resourceful in the whole of Oyo State. The reason is not far- fetched because the bulk of the Oyo State Industries lie within this Local Government area. [20] Ibadan South West Local Government has the largest concentration of industries and companies in the whole of Oyo State. About 50% of the Companies in the Local Government are located in Oluyole Industrial Estate while the remaining 50% spread across the Local Government area [20] Because of the concentration of industries, the inhabitants are highly enterprising.

With the use of the purely spatial scanning techniques for TB incidence, the LGA identified as the cluster with the highest risk for TB incidence was also Ibadan South-West and Ibadan North, using the Getis-Ord Gi* technique, which identifies hotspots, this local government area had a 99% confidence interval as a Hotspot area for TB incidence with the application of the space-time scanning technique also, Ibadan North and Ibadan South-West LGAs still maintained a high RR. These clusters can be used to map which LGAs need more resources to fight TB and meet up with the state’s as well as Nigeria’s target reductions in new cases of TB.

The high TB incidence observed could be a result of high case notification, high level of education of residents and availability of health care facilities and high socio-economic profile of the LGA. A study carried out on African countries by [21,22] supports this supposition stating that; Social and economic support was mentioned as a motivator for treatment adherence, ranging from promising strategies including regular SMS reminders, continuous, intense counseling, and financial incentives for the most underprivileged.

In the study by [22] in Kampala, the wealthiest sector of their study showed significantly lower odds of having an unsuccessful treatment outcome preceded by high notification rate and high level of health service availability and adherence.

The high TB incidence rate from our study could also be linked with the high population density in these local government areas, as these two LGAs are the most densely populated urban centers in Oyo state Nigeria. This agrees with a study by [23] which found the local region’s PTB notification rates were positively impacted by the population density of college students and health care costs per person in China, likewise, a study conducted by [24], in the democratic republic of Congo, based their study on the fact that areas with low population density have a lower TB incidence-rate compared to higher density areas.

A study conducted in Oyo state Nigeria found that the degree of variability in rates between urban and rural areas makes it imperative to survey rates of reported TB within smaller geographic units in order to pinpoint high-risk and low-risk zones that are not apparent at the state level [25]. To achieve this, using the Getis-Ord Gi* technique for TB incidence rates, we identified certain municipalities with spatial association, this generated hotspots and cold spots. Of these, Ido and Oluyole are classified as Hot Spots with 99% confidence interval. More importantly, these two LGAs share boundaries with each other.

Discovering specific areas in a geographical location with high or low TB loads provides insights for program planning and execution. This form of routine analysis, on geographically based data collected from time to time can be used to identify zones in a community where TB cases or risk factors for transmission are concentrated, in order to help public health workers, discern where community-based screening efforts may be directed as observed by a study in Kampala, Uganda [26]. Additionally, regions recognized as “cold spots” may indicate regions where diagnostic services may be lacking.

It is important to state that when it comes to analysis of spatial dependence, several factors come into play, some of them could be due to the care-seeking behaviors of the particular people in the population, or by access to and barriers to diagnostic and treatment facilities as evidenced by a study in Peru [27]. Data quality and the kind of geographical analytic approach employed may also have an impact on the spatial variation in reported TB rates. Our study for example used data collected manually at the Primary health centers DOTS facility from each LGA and collated at the state level during a period where passive case finding was the norm. These registries are therefore expected to miss a large number of TB cases and veer the observed clusters towards areas with better access to diagnostic services as observed with the rates reporting [1].

In males, the rate of TB cases was 15% higher than in females based on our result. Literature has established the prevalence of TB cases among males to be higher than females as seen in the research by [28] in Ethiopia who found that TB was more prevalent among adult males than among women even in a social context. Also, according to authors [1], higher proportion of TB cases among men is consistent with findings from prevalence surveys showing that TB disease affects men more than women and that gaps in case detection and reporting are greater among men, this could be because men tend to hide their sickness and do not frequent health facilities compared to women.

The incidence of TB varies by age group as well; the lowest TB incidence rate was seen in the 0–4 age group while the highest incidence rate was seen in the age group between 35–44. Largely, childhood TB incidence was low while incidence rate was higher among adults (age group 25–54). According to the [4], most often, adults with TB are those in their prime of life. All age groups are, nevertheless, at risk. According to authors [29], based on their study on a population in Shenzhen, age groups 25–34 years and 15–24 years, account for nearly 40% of total TB cases, being the age groups with the highest percentage of TB cases in their study, this corresponds with the current findings of our study. A study conducted in Kampala, Uganda, found that attributes surrounding age groups could be due to the high activity rate of individuals in this age bracket in search for means of livelihood and survival [1,26].

Regarding TB mortality, Ibadan Southwest has the highest mortality rate over the five years, which also has the highest incidence rate. This LGA is characterized by a relatively high development index in the entire state, which contributes to high case and mortality reporting in the state. According to the WHO [30] reports that areas with a high ratio of social determinants of health have high reports for TB occurrence. The non-medical factors that affect public health are referred to as social determinants of health (SDOH). In addition to the larger group of factors and systems influencing the quality of community life, being the conditions in which people are born, grow, work, live, and age [2,30].

It is also possible to see that the temporal trend for the incidence of TB in the period under study is decreasing; this could be due to low case detection in Nigeria as reported in the WHO global TB report and the fact that this was retrospective data and may have been due to information bias sequel to inadequate reporting [31]. In consonance with this, a study in Nigeria [32] found that due to an insufficient number of qualified officials, poor electricity output and scarcity of reagents for sputum smear examination, there was an inadequate functioning of diagnostic centers even at the primary and secondary health care centers in Nigeria, factors which all culminate to low case detection and inadequate reporting as experienced in Nigeria.

Another explanation to this could be a situation of generally improved health care service, in Oyo state which is a lesson and footstep set by the developed country. According to a study in developed Europe, [33], TB patients who visited TB facilities thought that the quality of access to care was excellent, that the attitude of the healthcare professionals was positive, that the facility’s appearance was excellent; that there were many people using the facilities, and that the wait time at a facility was under 30 minutes. This is consistent with a study conducted in the United States, where enhanced hygiene helped reduce the number of TB cases in the US, though rates continued to increase in poor, crowded neighborhoods. In addition, in Europe the TB burden in the WHO European Region as a whole is decreasing, and is down 19% overall for 2015–2019, according to the latest WHO/European Centre for Disease Prevention and Control (ECDC) report TB surveillance and monitoring in Europe 2021 [33]. Also, the first milestone of the End TB Strategy is a 20% reduction in the TB incidence rate (the number of new and relapse cases per 100 000 population per year) by 2020 compared with 2015 [1].

In Fig 5B we see a slight increase in the TB mortality rate throughout the analyzed period, we can also see that the temporal trend of mortality between 2015 and 2019 is increasing.

The End TB Strategy of the WHO outlines global goals and targets for significant decreases in the annual number of TB deaths between 2016 and 2035. The first goal is a decrease of 35% from 2015 to 2020. Following plans for reductions of 90% by 2030 and 95% by 2035, the next milestone in 2025 is a 75% decrease from 2015 levels [3]. It is necessary to accomplish worldwide milestones and targets for reducing the number of people who contract TB each year in order to meet these targets. The slight increase in the mortality rate observed could be a sequel to this set target by the WHO in a bid to reduce the burden. Improved case reporting and surveillance would increase the number of cases reported and point out the fatality of TB for a more effective control.

As limitations of the present investigation, it is important to note that this is an ecological study, which means that we must recognize the presence of the so-called ecological fallacy. In this type of study, when using data at the aggregate level, the results may not directly reflect associations at the individual level, highlighting the need to carry out more research with different designs and methods to draw more accurate and reliable conclusions. Additionally, it is important to consider the use of secondary data sources, which can lead to incomplete information (such as undiagnosed cases) or typing errors, which can affect the results, with incidence and mortality rates being underestimated or overestimated.

Regarding the secondary data used in the present study, it is important to mention the scarcity of information on TB cases, as Nigeria faces problems of underreporting due to several reasons, including lack of access to health services, inadequate diagnosis, and lack of infrastructure. adequate healthcare in rural and remote areas. Furthermore, lack of financial, technological, and human resources limits the ability of the Nigerian health system to adequately collect, store and analyze data on TB It is also important to mention the fragmentation of the Nigerian healthcare system that can result in gaps in data collection and sharing between different healthcare agencies and institutions, leading to a lack of consolidated and accessible data on TB.

In view of this, an important limitation of this study was the period of available notifications (2015–2019) and which were only made available to us on an annual basis, which was an impediment to carrying out more in-depth analyses, mainly regarding the seasonality of the disease (which would be better explored if monthly data were available. Addressing these issues is important to improve TB surveillance and develop effective strategies for preventing and controlling the disease and, therefore, it is suggested that other studies with new approaches be carried out with more complete notification data, in order to understand the dynamics of the disease in African countries.

Another limitation of the study that should be mentioned is the use of population-based data obtained from (https://citypopulation.de/en/nigeria/admin/NGA031__oyo/) to calculate rates. Due to this lag, demographic data may not fully reflect the population reality during the study period, which can potentially impact the analyses conducted.

## Conclusions

In conclusion, the results of the present study contributed to knowledge by highlighting Local government Areas in Oyo state Nigeria with high TB risk and rates between 2015–2019, the spatial and temporal distribution and areas with higher risk of TB transmission. This makes it possible for policy makers to target hotspot areas for intervention and disease prevention. We recommend that further in-depth studies regarding spatial analysis should be developed in Oyo state.

## Supporting information

S1 Table Clinical and epidemiological profile of TB cases in Oyo state Nigeria. (XLS)

S2 Table Sociodemographic profile of cases in Oyo State, Nigeria. (DOCX)

S1 File Ethical Approval by the Oyo State Research and Ethics 1 Committee Board of the Ministry of Health, Oyo State, Nigeria.
(PDF)

S1 Fig Decompose of time series of tuberculosis incidence and mortality rates, Oyo – Nigeria (2015–2019). (TIF)
