# Supplementary material for: Surveillance of tuberculosis incidence and mortality through spatio-temporal analysis in Oyo State, Nigeria
Source: PLoS One. 2025 Jul 16;20(7):e0311739. doi: 10.1371/journal.pone.0311739 (PMC12266401; doi:10.1371/journal.pone.0311739)
Supplement: S2 Table — (DOCX) [file pone.0311739.s002.docx]

**S2 Table.** Sociodemographic profile of cases in Oyo State, Nigeria

| **2015** | | | | | | | | | | | | | | | | | | | | | | | | | | | | | | | | |
| --- | --- | --- | --- | --- | --- | --- | --- | --- | --- | --- | --- | --- | --- | --- | --- | --- | --- | --- | --- | --- | --- | --- | --- | --- | --- | --- | --- | --- | --- | --- | --- | --- |
| **Sex** | **0-4** | | | **5-14** | | | | **15–24** | | | | **25–34** | | | | **35–44** | | | **45–54** | | | | **55–64** | | | | **> 65** | | | | | **Total** |
| **Male** | 63 | | | 91 | | | | 424 | | | | 978 | | | | 926 | | | 647 | | | | 373 | | | | 340 | | | | | 3842 |
| **Female** | 60 | | | 112 | | | | 431 | | | | 793 | | | | 630 | | | 392 | | | | 225 | | | | 213 | | | | | 2856 |
| **Total** | 123 | | | 203 | | | | 855 | | | | 1771 | | | | 1556 | | | 1039 | | | | 598 | | | | 553 | | | | | 6698 |
| **2016** | | | | | | | | | | | | | | | | | | | | | | | | | | | | | | | | |
| **Sex** | **0-4** | | **5-14** | | | | **15–24** | | | | **25–34** | | | | **35–44** | | | | | **45–54** | | | | **55–64** | | | | **> 65** | | | **Total** | |
| **Male** | 59 | | 107 | | | | 420 | | | | 896 | | | | 909 | | | | | 705 | | | | 400 | | | | 333 | | | 3829 | |
| **Female** | 46 | | 108 | | | | 416 | | | | 724 | | | | 666 | | | | | 407 | | | | 249 | | | | 219 | | | 2835 | |
| **Total** | 105 | | 215 | | | | 836 | | | | 1620 | | | | 1575 | | | | | 1112 | | | | 649 | | | | 552 | | | 6664 | |
| **2017** | | | | | | | | | | | | | | | | | | | | | | | | | | | | | | | | |
| Sex | 0-4 | 5-15 | | | 15–24 | | | | | 25–34 | | | 35–44 | | | | 45–54 | | | | 55–64 | | | | > 65 | | | | Total | | | |
| **Male** | 55 | 123 | | | 413 | | | | | 945 | | | 1070 | | | | 759 | | | | 379 | | | | 341 | | | | 4085 | | | |
| **Female** | 60 | 116 | | | 404 | | | | | 694 | | | 703 | | | | 423 | | | | 214 | | | | 202 | | | | 2816 | | | |
| **Total** | 115 | 239 | | | 817 | | | | | 1639 | | | 1773 | | | | 1182 | | | | 593 | | | | 543 | | | | 6901 | | | |
| **2018** | | | | | | | | | | | | | | | | | | | | | | | | | | | | | | | | |
| **Sex** | **0-4** | | **5-15** | | | **15–24** | | | **25–34** | | | | | **35–44** | | | | **45–54** | | | | **55–64** | | | | **> 65** | | | | **Total** | | |
| **Male** | 58 | | 82 | | | 401 | | | 873 | | | | | 1080 | | | | 779 | | | | 361 | | | | 441 | | | | 4075 | | |
| **Female** | 43 | | 112 | | | 365 | | | 695 | | | | | 664 | | | | 429 | | | | 280 | | | | 257 | | | | 2845 | | |
| **Total** | 101 | | 194 | | | 766 | | | 1568 | | | | | 1744 | | | | 1208 | | | | 641 | | | | 698 | | | | 6920 | | |
| **2019** | | | | | | | | | | | | | | | | | | | | | | | | | | | | | | | | |
| **Sex** | **0-4** | | **5-14** | | | **15–24** | | | **25–34** | | | | | **35–44** | | | | **45–54** | | | | **55–64** | | | | **> 65** | | | | **Total** | | |
| **Male** | 15 | | 30 | | | 90 | | | 209 | | | | | 276 | | | | 206 | | | | 115 | | | | 129 | | | | 1070 | | |
| **Female** | 18 | | 35 | | | 83 | | | 136 | | | | | 197 | | | | 124 | | | | 78 | | | | 73 | | | | 744 | | |
| **Total** | 33 | | 65 | | | 173 | | | 345 | | | | | 473 | | | | 330 | | | | 193 | | | | 202 | | | | 1814 | | |
