# Supplementary material for: Surveillance of tuberculosis incidence and mortality through spatio-temporal analysis in Oyo State, Nigeria
Source: PLoS One. 2025 Jul 16;20(7):e0311739. doi: 10.1371/journal.pone.0311739 (PMC12266401; doi:10.1371/journal.pone.0311739)
Supplement: S1 File — Ministry of Health, Oyo State, Nigeria. (PDF) [file pone.0311739.s003.pdf]

S1 Fig -Ethical Approval by the Oyo State Research and Ethics Committee Board of the Ministry of Health, Oyo State, Nigeria

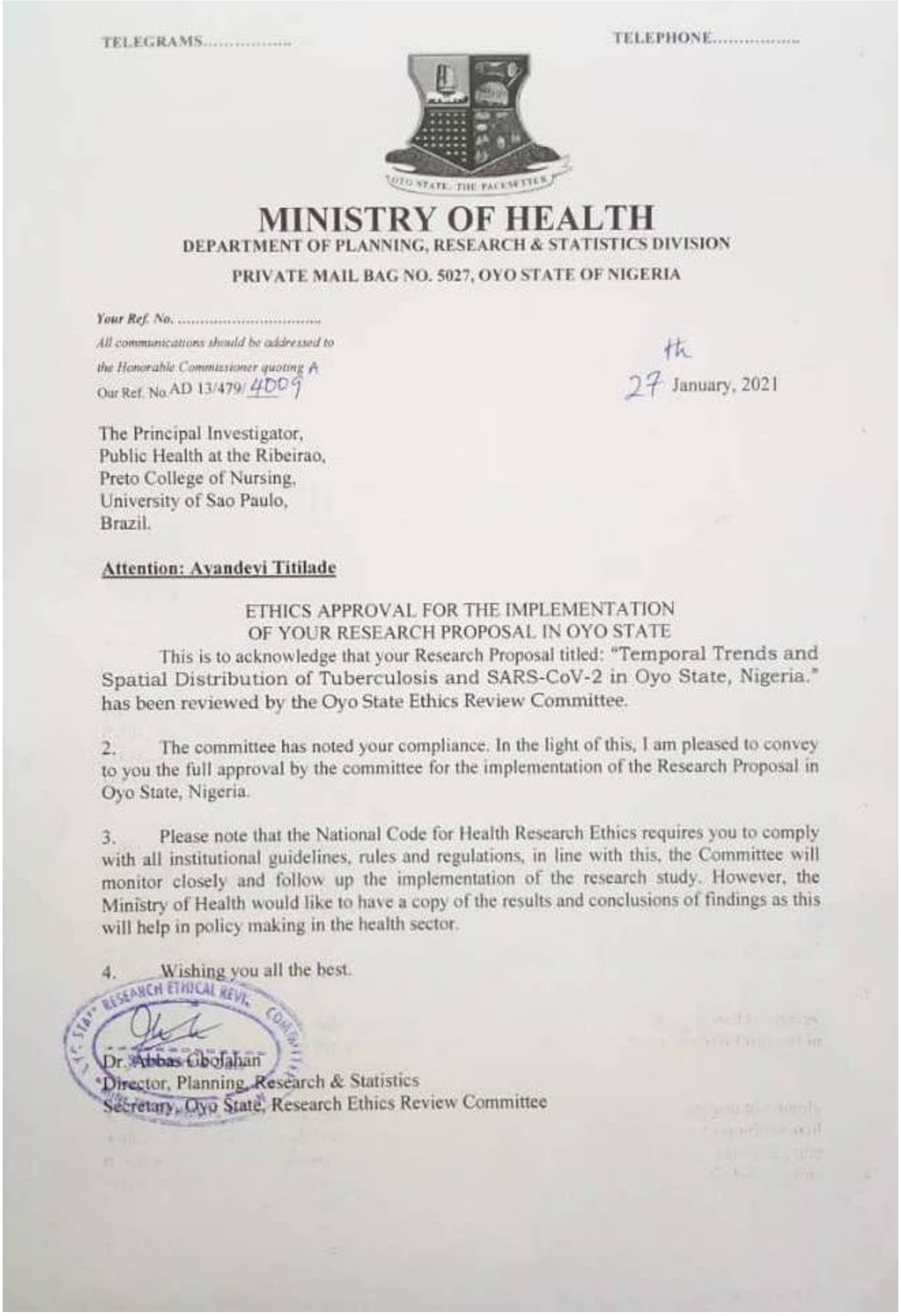

TELEGRAMS................ TELEPHONE................

OYO STATE. THE PACESETTER

MINISTRY OF HEALTH

DEPARTMENT OF PLANNING, RESEARCH & STATISTICS DIVISION

PRIVATE MAIL BAG NO. 5027, OYO STATE OF NIGERIA

Your Ref. No. ....

All communications should be addressed to the Honorable Commissioner quoting A

Our Ref. No. AD 13/479/ 4009

27th January, 2021

The Principal Investigator,
Public Health at the Ribeirao,
Preto College of Nursing,
University of Sao Paulo,
Brazil.

Attention: Ayandeyi Titilade

ETHICS APPROVAL FOR THE IMPLEMENTATION
OF YOUR RESEARCH PROPOSAL IN OYO STATE

This is to acknowledge that your Research Proposal titled: "Temporal Trends and Spatial Distribution of Tuberculosis and SARS-CoV-2 in Oyo State, Nigeria." has been reviewed by the Oyo State Ethics Review Committee.

2. The committee has noted your compliance. In the light of this, I am pleased to convey to you the full approval by the committee for the implementation of the Research Proposal in Oyo State, Nigeria.

3. Please note that the National Code for Health Research Ethics requires you to comply with all institutional guidelines, rules and regulations, in line with this, the Committee will monitor closely and follow up the implementation of the research study. However, the Ministry of Health would like to have a copy of the results and conclusions of findings as this will help in policy making in the health sector.

4. Wishing you all the best.

Dr. Abbas Gbolahan
Director, Planning, Research & Statistics
Secretary, Oyo State, Research Ethics Review Committee
