# Supplementary figures and images for: Surveillance of tuberculosis incidence and mortality through spatio-temporal analysis in Oyo State, Nigeria
Source: PLoS One. 2025 Jul 16;20(7):e0311739. doi: 10.1371/journal.pone.0311739 (PMC12266401; doi:10.1371/journal.pone.0311739)

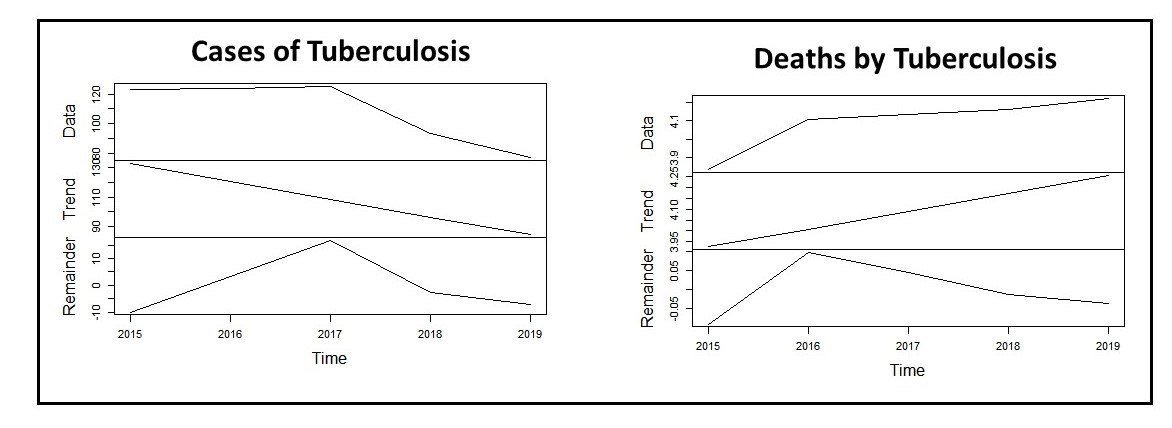

Supplement: S1 Fig — (TIF) [file pone.0311739.s004.tif]
